# I Had to Go in a Bubble: Investigating the Effects of COVID-19 on Fertility Treatments and Nutrition

**DOI:** 10.1089/whr.2022.0028

**Published:** 2022-06-29

**Authors:** Elizabeth A. Claydon, Diana L. Davidson, Kathleen M. McCarty, Jeffrey Wang

**Affiliations:** ^1^ Department of Social and Behavioral Sciences, West Virginia University School of Public Health, Morgantown, West Virginia, USA.; ^2^ Department of Sociology, West Virginia University Eberly College of Arts and Sciences, Morgantown, West Virginia, USA.; ^3^ Child Health Research Center, The University of Queensland, Queensland, Australia.; ^4^ American Fertility Services, Greenwich, Connecticut, USA.

**Keywords:** fertility, infertility, COVID-19, food access, social support, nutrition

## Abstract

**Background:**

Individuals modify their lifestyles including nutrition to improve fertility. The COVID-19 pandemic limited access to clinical offices or resulted in the closure of fertility clinics. The pandemic also impacted diet through reduced availability and lifestyle choices. This article's purpose was to understand the consequences of the COVID-19 pandemic on diet, lifestyle, and the course of fertility treatment.

**Methods:**

The research was conducted through qualitative data collected for a larger study regarding fertility and nutritional guidance. Thematic analysis from eight interviews was used to uncover major and subthemes among the transcripts.

**Results:**

The results showed two resulting main themes: disruption and distress on the fertility journey of these individuals, as well as the added stress of limited food access, which reduced their ability to continue their dietary choices.

**Conclusions:**

Our findings indicate substantial disruptions to food access and to individuals' fertility treatment during the first year of the COVID-19 pandemic. Recommendations from these findings suggest that consistent clinic policies can allow for increased support system integration. A further recommendation is the need for a multidisciplinary team to support the individuals going through fertility treatments, such as a registered dietitian to help meal plan around their diet protocol. A registered dietitian would be able to assist patients in making adjustments when faced with limited access to certain food resources as a result of the pandemic.

## Introduction

Being pregnant during COVID is bad enough, but when you add all of the immune suppressing meds it's extremely dangerous. I have a child and I have an obligation to be there and not to put my life at risk to have a second. ∼ Sally^*^

The novel severe acute respiratory syndrome coronavirus 2 (SARS-CoV-2, which causes the disease COVID-19) was first detected in December 2019 and on March 11, 2020 the World Health Organization declared a global pandemic.^1^ The U.S. government implemented protocols of social distancing, mandating masks to be worn in public gatherings, and resulted in the reduced capacity or closure of multiple institutions, including fertility clinics.^2^

*In vitro* fertilization (IVF) is a common assisted reproductive technology (ART) and has steadily increased its utilization since the first successful birth in 1981.^3^ It is estimated that 1.8% of infants in the United States are conceived annually through ART. However, there was a significant drop in the practice of fertility services during the COVID-19 pandemic as a result of the guidelines provided by the American Society for Reproductive Medicine (ASRM).^3^ To further support this, a study done by Strata Decision Technology of 228 hospitals across 40 states yielded results that showed encounters for infertility services were down 83% from March 22 to April 4, 2020 in comparison with the year prior.^3^

The largest priority for pregnant women is to avoid contracting COVID-19 due to potential pregnancy complications. The effect of COVID-19 was unknown at the start of the pandemic, but it is well established that pregnant women are more susceptible to respiratory infection.^4^ Pregnant women were more likely to be hospitalized and be placed in the intensive care unit; in addition, there was a 2% increase in pregnancy loss and a slight increase in preterm deliveries.^5–7^ It is undetermined whether these increases are due solely to SARS-Cov-2 infection, but may be due to indirect effects such as access, stress, or staffing shortages.^6^

Although there is limited information available on the effects of COVID-19, there is a known risk of high fever associated with COVID-19 symptoms that can raise the risk of birth defects, which is most critical during the first trimester. These risks explain the significant precautions and restrictions being imposed at the current time of the study at fertility clinics and doctor offices across the country. Many clinics closed during stay-at-home orders due to both patient and medical staff safety, consequently delaying IVF cycles and transfers.^8^

In response to the unprecedented nature of the transmission and infection rate of COVID-19, the U.S. response to reproductive care and clinics was coordinated by the ASRM.^9^ The ASRM issued guidelines that were intended to prevent health care-acquired infections, as well as limit the usage of health care resources on nonurgent treatment. Therefore, it was recommended to suspend initiation of new fertility treatment cycles, such as intrauterine insemination (IUI), IVF, and egg retrieval procedures. It is important to note that the ASRM task force did consider the time-sensitive nature of fertility treatment when creating these guidelines.

One study suggested that half of participants disagreed with the suspension of treatment and restricted guidelines in place as a result of the pandemic.^2^ Participants would have preferred to resume their treatment despite there being little knowledge about the effect of COVID-19 on pregnancies and increasing rates of infection. The same study concluded that during the beginning of the shutdown due to the COVID-19 pandemic, there was an association with the female patient's negative emotional reactions.

The suspension of fertility treatment had accounted for 86% of participants reporting a negative impact on their mental health. However, the study revealed that women who were surrounded with a strong emotional support system from their partner and family were reporting smaller negative mental health impacts in comparison with women who were seeking treatment for a long period of time.^10^

The focus of this article is to understand from the lived experience of women going through infertility treatment and the impact of COVID-19 on their fertility treatment and dietary lifestyle adjustments they had to make as a result of the ongoing pandemic. This study utilized a transcendental phenomenological framework to understand the essence of the fertility experience from those who live it.^11^ In this way, we aimed not only to understand objectively fertility treatments during COVID-19, but their perception of experiencing fertility treatments during COVID-19.

## Methods

### Positionality statement

In all research, but particularly in qualitative research, it is imperative that the researchers have a strong awareness of their position and context in relation to the topic they are studying. Therefore, as authors, we offer a positionality statement that provides some of this background. E.C. was the primary investigator in this study and has one child but has not experienced fertility treatments. E.C. has a PhD in public health and researches eating disorders, disordered eating, body image, and the intersection between eating and pregnancy, which drew her to explore eating behaviors in relation to fertility treatments. She has had extensive experience interviewing and reporting on other qualitative topics, including those related to women's mental and perinatal health. D.D. was a student researcher in sociology at the time of this research, interested in maternal and child health, but without children of her own. K.M. has an ScD in epidemiology and came up with the research question as a result of her own IVF child. She has two children, one through IVF and participated in the patient support group for diet and IVF. J.W. is a physician practicing reproductive endocrinology/infertility and has one child, but has not experienced fertility treatments.

### Human subjects

Approval was obtained from West Virginia University's Institutional Review Board (IRB) (IRB No. 2001854867) for this research. The inclusion criteria for the participants were as follows: English-speaking, women (age 18 and older) currently undergoing IVF toward egg retrieval or who have gone through the IVF process in the past. Once participants volunteered and consented to be a part of our research, they were interviewed by E.C. through Health Insurance Portability and Accountability Act-protected zoom calls to discuss their IVF journey, sources of guidance and nutrition, and the outcome of the effect of the diet on them.

### Recruitment

Women were purposively recruited through an online fertility support group from an East Coast fertility clinic through an introduction by K.M. and approval granted to E.C. to advertise for recruitment. A short advertisement, including eligibility criteria and an e-mail to contact, was posted on the group before interviews; all participants completed a short demographic questionnaire through e-mail. Once participants were scheduled through e-mail, they were asked in advance if interviews could be recorded and all agreed. D.D. took extensive fieldnotes during each interview.

### Data analysis

Data analysis was based on verbatim transcripts of audio recordings and Nvivo 11 was used to manage and code transcript data to compile major and subthemes. Transcripts were coded by E.C. iteratively according to guiding principles *in vivo* and based on best descriptors of text. A second coder (D.D.) reviewed >20% of the total sample after being trained on the coding system; this proportion is adequate based on Loewen and Plonsky's recommendation.^12^ Discrepancies between on codes during training were resolved during discussions between the coders and allowed for a final coding structure to emerge. Transcripts of the interviews were de-identified before analysis and pseudonyms were used employing a random name generator to preserve confidentiality yet allow for personalization of quotes.

The data for this research article were gathered over a short span between November 2020 to January 2021. This was before the resurgence of the third and fourth wave of COVID-19 and the identification of other variants, such as the Delta and Omicron.^1^

### Participants

There were a total of 10 women interviewed over a 3 month span of time for a larger study regarding fertility and nutrition. A total of 11 women initially contacted the e-mail in the advertisement to schedule an interview and 10 out of the 11 completed the interviews. One did not complete the interview due to time constraints and being in the middle of fertility treatments. Of those 10 completed interviews, 7 had experienced either fertility processes or pregnancies that were affected by the COVID-19 pandemic and were included in this analysis. The average age was 37.1 ± 7.67. Thirty percent had an income of >150 k per year and 71.4% had a full-time job. All had at least a college education, were married, heterosexual, White, non-Hispanic, and had private insurance. The average number of children to women in the sample was 1 ± 0.82. See demographic descriptions in Table 1.

**Table 1.** Demographic Characteristics of Participants

Name^a^	Carol	Joann	Macy	Kaylee	Madeline	Katelyn	Sally
Age	36	53	35	30	34	32	40
Average income	Prefer not to say	150–249 k	150–249 k	50–149 k	50–149 k	50–149 k	150–249 k
No. of children	1	2	0	1	2	0	1
Working status	Full time	Disability	Full time	Seeking employment	Full time	Full time	Full time
Education	Graduate school	College	Graduate school	College	College	Graduate school	College

^a^ Pseudonyms to protect participant identity.

## Resulting Themes

From the thematic analysis, two themes around COVID-19 emerged: the *Impact of COVID-19 on IVF Journey* and *Impact of COVID-19 on Food Access.* These themes are discussed hereunder with representative quotes from the participants to elucidate their lived experience in their own words. Table 2 details the definitions of these two main themes.

**Table 2.** Themes

Theme/subtheme	Description
Impact of COVID-19 on IVF journey	Any mention of the role COVID-19 had on affecting participant IVF journey, including restrictions on partner support at appointments, halts to egg retrievals, and other appointments.
Impact of COVID-19 on food access	Discussions around the impact of COVID-19 on participant ability to adhere to the diet standards that they were adopting during their IVF journey.

IVF, *in vitro* fertilization.

### Impact of COVID-19 on IVF journey

COVID-19 has impacted the experience of fertility treatment and altered the timelines of families seeking treatment by delaying it. There were several modifications that facilities had to make to accommodate the guidelines that the CDC placed to prevent the spread of the virus. This can be in the form of no guests allowed during visits, halt on any further procedures, and so forth.

For example, some women discussed the challenge of fertility appointments and egg retrievals without the support of their partners due to COVID-19 restrictions. Katelyn spoke to this issue when stating:

the hardest impact that [COVID-19] had is that partners aren't allowed to go to appointments…. someone who's had recurrent pregnancy loss … there is an incredible amount of Post-Traumatic Stress Disorder associated with ultrasounds. I can't go into an ultrasound room without having a panic attack, it's just impossible…Thankfully, in our case, we have been able to talk to the higher ups at our hospitals and I have gotten exceptions for my husband to be there.

Not having their partner seemed to take an emotional toll on participants because they relied even more heavily on that support system based on past traumatic experiences with fertility and pregnancy. Going to appointments was, therefore, an event that both not only looked forward to doing together but also felt the importance of that partner's support in that emotional setting; however, one partner was often left out on the journey. Katelyn continued to describe her journey and the absence of her husband before accommodations being made as being upsetting because “him not being there for [egg retrievals] you know it was still sad like you know you get to see the embryo implant and you know he never will.”

In the beginning of the pandemic, the population was unaware of the potential impact COVID-19 would impose on pregnancies when contracted. Therefore, it caused stress on families who were trying to conceive or who were already pregnant. One participant, Madeline, discusses the effects by stating:

I was probably about thirty-five weeks at that point so it was terrifying, you know, we didn't know what COVID-19 would do to pregnant women. We didn't know what COVID would do to babies *in utero*. So, I had to go in a bubble. I was only leaving the house for doctor's appointments.

Others also expressed some of the challenges related to isolation from friends and family during the pandemic in relation to their IVF journey. Joann said: “It's a scary time. You know I even have friends and family who … after fighting years for my second child … have only met them on Facebook.”

When thinking about future retrievals, Joann and others felt similarly about her decision to keep her family and self safe by through quarantining as a family for the pregnancy and most of her child's first year. “I will not put myself at risk to get the virus. No matter what my friends who are nonbelievers say or whatever the case is. And I can't put my husband at risk. I can't put my children at risk. It's just not worth it.”

### Impact of COVID-19 on food access

COVID-19 had greatly impacted the resources and food availability when it first hit in March 2020.^13^ It restricted the ability to access certain food groups, some fresh produce, as well as saw a slight rise in prices. This greatly impacted some participants' ability to adhere to their diet and they had to make some adjustments to accommodate this problem. Therefore, participants often discussed the frustration that they felt and the different ways they would have to rework recipes or adapt their diets.

Sally gave light to this when speaking about how “the first month it was impossible to get any grocery deliveries, we couldn't get anything. I mean we put a delivery for 50 items, and I would get 5 so we have no fresh vegetables and no fresh fruits. So, I went out and got a deep freezer and the two refrigerators … so, we have food, but we didn't have any fresh food. So, it got to the point where it was like … am I going to be eating frozen vegetables for the next six months?” In a situation where women are being very careful with their food and nutritional intake with fertility as an end goal, this is often a very challenging adaptation to make.^14^

COVID-19 impacted the ability of some women to even go shopping for grocery items because of their doctor's protocol to stay home and avoid going out into public unless it was necessary. This caused complications for women to continue to follow diet protocols and have control over both quality and type of food selected, especially when relying on third parties. Madeline spoke to this issue as she stated:

I was no longer able to go and select my produce and my meat. I had to rely on other people, Instacart, and you know different grocery delivery options and quite frankly I don't know that the quality was as good as what I would have personally picked, but you know, we all kind of just had to do our best.

Not only did COVID-19 affect the quantity and quality of food selection, but participants stated they needed to make adjustments to how they meal prep for the week and shopping habits. During COVID-19 it was encouraged that the population refrain from venturing out often into the public to help lessen the chance of transmission of the virus. Therefore, people would try lessen their frequency of grocery shopping by getting what they needed all at once to make it last for a longer time. One participant, Kaylee, highlighted this issue by stating:

Going to the grocery stores … fresh food is not always in the cards for us. So, it's a little difficult to manage making sure I do a lot of meal planning so that I can make sure that that one trip every two weeks I can get just about everything I need … it's been really difficult, but we've been trying to manage it

Overall, the impact of COVID-19 affected individuals' lifestyle by restricting access to food options due to shortages, making adjustments to how people shop and prepare their food for the week. It caused undue stress to women and families who were seeking conception by adding another factor they needed to be cautious about to prevent any complications to their pregnancy journey.

## Discussion

Our study shows similar reactions to the Marom Haham et al.^2^ study, as many women had a desire to continue treatment and showed signs of distress when their treatments were restricted. In addition, our findings expanded on this, indicating the COVID-19 restrictions on the number of individuals present at appointments could have resulted in a lack of partner and/or family support for these women.

However, from the quotes provided these women also expressed the general uncertainty of safety for pregnancy during COVID, especially for individuals who were immune-suppressed. Carol spoke about how she tried to “focus on the present and trust that when that time comes, things will get handled” and stated that “I haven't really let my mind go to like okay you're going to be pregnant on an autoimmune protocol during a pandemic.” There was a general anxiety around continuing fertility treatment during the pandemic, as well as different coping mechanisms for addressing those fears.

As part of this fertility journey, some individuals had to put egg retrievals on hold due to COVID-19. Some individuals chose to pause fertility treatments in addition to clinic restrictions^8,9^ due to the risk. Others were impacted by the restrictions or were unsure what would change in the next few months as they continued their fertility process. The variable nature of the pandemic throughout its waves made planning a course challenging, and this unpredictable nature may have influenced their experience of it. Macy mentioned “nothing has been impacted yet, knock on wood,” although she also indicated a change based on her husband not being able to come into the clinic for her second and third retrievals. However, although her retrievals were relatively unaffected, her uncertainty about the future is an indication of the feelings of the time.

From a phenomenological standpoint, the essence of these individuals' experience can be reflected succinctly by the phrase provided by a participant in the title: “I had to go in a bubble.” Women were not only often isolated from their social support systems when they needed them the most at fertility appointments, but also were constrained from freely choosing foods in line with nutritional guidance for their fertility treatments. This isolating and restricting experience made women feel constrained in choices, and often making the best of the situation that they could. However, all of these women were also recruited from an online support group that provided a sense of social support, camaraderie, and source of connection in a time of general physical isolation.

### Limitations

Through the analysis of information gathered from the demographic questionnaire on the participants, it was shown that there were no participants who were people of color or from a lower socioeconomic class. Another limitation to be aware of is that the information and data collected for this research was during some of the height of the COVID-19 pandemic. Since the completion of this research, the pandemic has continued to rage on and changed drastically. These data give a snapshot of COVID-19 from its discovery in December 2020 and before the vaccine was available to the general public.

### Strengths

This is the first study that we are aware of to qualitatively research the impacts of COVID-19 on the fertility process, including aspects of food access. This lived experience has the potential to inform clinicians regarding some of the challenges and adaptations their patients have had throughout the pandemic.

## Conclusion

From our findings, we suggest two possible solutions to help improve overall quality in fertility treatment while also following protocols put in place by clinics and higher institutions during the pandemic. The first is to institute consistent policies across clinics to allow for integration of a support system. This could include having one partner or chosen person present so they can have a sense of security as well as a support system during the process. The psychological impact of the restrictions and suspensions of fertility treatment is evident throughout past and current studies as an abundance of women reported feeling overwhelmed and had a strong desire to have a partner with them during treatment.

Our interviews indicated that clinics made adjustments for some patients to allow their partner to come into appointments, whereas others were strict and made no such effort; often, these decisions were on a case-by-case basis. Other research is indicating the importance of heightened communication between fertility clinics and patients as well as the use of online platforms to reduce some of these feelings of isolation and distress due to fertility disruptions from the pandemic.^15^

The second recommendation is to alleviate the consequences of the pandemic during fertility treatment, there may be a need for a multidisciplinary team to support patients. One such way is adding a registered dietitian (RD) to work with patients on how to meal plan around their diet protocol and how to make proper adjustments when faced with limited access to certain food resources. Many women in our study reported mild to severe limitations in their continuation of following their diet protocols because of the pandemic with a few halting their fertility diets altogether and alternating for a different one.

## Data Availability

Data are available on reasonable request from the authors.

## Abbreviations Used

ART: assisted reproductive technology
ASRM: American Society for Reproductive Medicine
IVF: *in vitro* fertilization
IUI: intrauterine insemination
SARS-CoV-2: severe acute respiratory syndrome coronavirus 2

## Notes

^*^
Pseudonyms used to protect participants' identities.
